# Targeting Mitochondrial-Derived Reactive Oxygen Species in T Cell-Mediated Autoimmune Diseases

**DOI:** 10.3389/fimmu.2021.703972

**Published:** 2021-07-01

**Authors:** Miranda D. Chávez, Hubert M. Tse

**Affiliations:** Department of Microbiology, Comprehensive Diabetes Center, University of Alabama at Birmingham, Birmingham, AL, United States

**Keywords:** autoimmunity, T cell, immunometabolism, mitochondria, reactive oxygen species

## Abstract

Mitochondrial dysfunction resulting in oxidative stress could be associated with tissue and cell damage common in many T cell-mediated autoimmune diseases. Autoreactive CD4 T cell effector subsets (Th1,Th17) driving these diseases require increased glycolytic metabolism to upregulate key transcription factors (TF) like T-bet and RORγt that drive differentiation and proinflammatory responses. However, research in immunometabolism has demonstrated that mitochondrial-derived reactive oxygen species (ROS) act as signaling molecules contributing to T cell fate and function. Eliminating autoreactive T cells by targeting glycolysis or ROS production is a potential strategy to inhibit autoreactive T cell activation without compromising systemic immune function. Additionally, increasing self-tolerance by promoting functional immunosuppressive CD4 T regulatory (Treg) cells is another alternative therapeutic for autoimmune disease. Tregs require increased ROS and oxidative phosphorylation (OxPhos) for Foxp3 TF expression, differentiation, and anti-inflammatory IL-10 cytokine synthesis. Decreasing glycolytic activity or increasing glutathione and superoxide dismutase antioxidant activity can also be beneficial in inhibiting cytotoxic CD8 T cell effector responses. Current treatment options for T cell-mediated autoimmune diseases such as Type 1 diabetes (T1D), multiple sclerosis (MS), rheumatoid arthritis (RA), and systemic lupus erythematosus (SLE) include global immunosuppression, antibodies to deplete immune cells, and anti-cytokine therapy. While effective in diminishing autoreactive T cells, they can also compromise other immune responses resulting in increased susceptibility to other diseases and complications. The impact of mitochondrial-derived ROS and immunometabolism reprogramming in autoreactive T cell differentiation could be a potential target for T cell-mediated autoimmune diseases. Exploiting these pathways may delay autoimmune responses in T1D.

## Introduction

**Figure 1:**
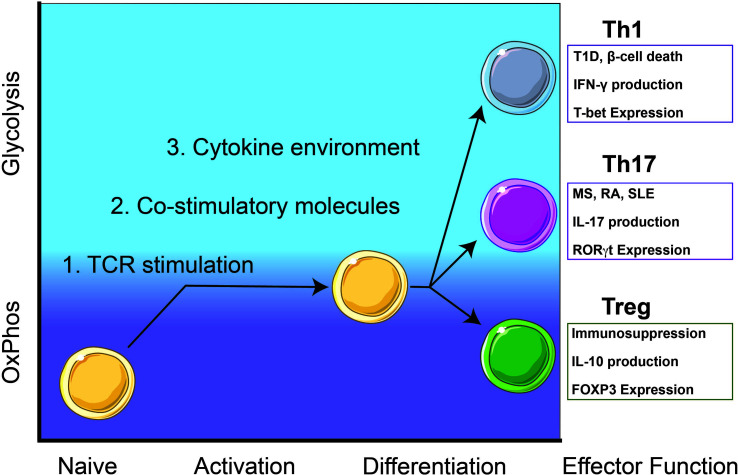
**Autoreactive Th1 and Th17 T cell responses rely on glycolysis while immunosuppressive Treg cells utilize oxidative phosphorylation (OxPhos).** Th1 cells contribute to β-cell death and destruction in type 1 diabetes (T1D) and Th17 cells contribute to pathogenesis of other T-cell mediated diseases including multiple sclerosis (MS), rheumatoid arthritis (RA), and systemic lupus erythematosus (SLE). Upon activation, naïve T cells will metabolically shift from OxPhos-dependence to a balance between glycolysis and OxPhos. Throughout early activation and differentiation, this balance is maintained until a commitment toward an effector function is achieved. Once fully differentiated, autoreactive Th1 and Th17 cells utilize glycolysis for homeostasis and maintenance while immunosuppressive Treg cells rely on OxPhos.

Failure to maintain self-tolerance leads to autoreactive T cells that recognize systemic or organ-specific self-antigens and subsequently, the development of autoimmunity (1). Mechanisms that result in decreased regulatory T cell (Treg) numbers and/or function could be contributing to self-tolerance failure. Tregs are essential in maintaining self-tolerance by secreting immunosuppressive/anti-inflammatory cytokines including IL-10 and TGF-β, and expression of the inhibitory receptor, CTLA-4 (2, 3) (**Figure 1**). Re-establishing self-tolerance by increasing Treg cell numbers and/or increasing Treg immune suppression function may inhibit autoreactive T cell effector responses and delay the progression of T cell-mediated autoimmune diseases (4). CD4 T cell subsets associated with T cell-mediated autoimmune diseases include T helper (Th) Th17 and Th1 cells.

Generally, Th17 cells mediate immunity to extracellular pathogens and are characterized by the expression of the transcription factor (TF) RORγt and the production of cytokines such as interleukin (IL)-17A/F (5) (**Figure 1**). Th17 cells are involved in autoimmune-mediated diseases such as multiple sclerosis (MS), rheumatoid arthritis (RA), and systemic lupus erythematosus (SLE) (6, 7) (**Figure 1**). Th1 T cell responses are responsible for cell-mediated immunity and are characterized by the expression of the TF T-bet and the production of IFN-γ (8) (**Figure 1**). In Type 1 diabetes (T1D), CD4 T cell Th1 cytokine responses and CD8 cytotoxic T cell responses contribute to inflammation and destruction of insulin-producing β-cells (9) (**Figure 1**).

Current strategies to treat T cell-mediated autoimmune diseases include the use of low dose IL-2, mTOR inhibition (rapamycin), T cell depletion antibodies (teplizumab, anti-thymocyte globulin), or cytokine neutralizing antibodies/soluble receptor proteins (Infliximab, Etanercept) (10–12). These treatments are successful in temporarily diminishing disease pathogenesis; however, these reagents do not provide a permanent cure for autoimmune diseases. Therefore, further research is required to fully understand the mechanisms involved in autoreactive T cell differentiation in autoimmune disease development. One mechanism that is involved in T cell fate and function includes cellular metabolism and specifically, the interplay between signaling pathways involved in T cell differentiation and metabolic reprogramming to determine T cell effector responses (13, 14).

Immunometabolism has garnered extensive attention in recent years due to the reliance of specific metabolic pathways necessary for efficient T cell activation and differentiation of T cell subsets. Immunometabolic pathways of interest includes glycolysis, oxidative phosphorylation (OxPhos), and the contribution of mitochondrial-derived reactive oxygen species (mtROS) to mediate autoreactive T cell activation and differentiation by functioning as signaling molecules (7, 15, 16). Therefore, reprogramming T cell metabolism by targeting mtROS may counteract autoreactive T cell function and proinflammatory responses (17). This review will discuss the impact of mtROS and its potential target for immunometabolism reprogramming therapy in the context of T cell-mediated autoimmune diseases.

## Activation of Naïve CD4 T Cells Are Primed for Differentiation by Shifting Metabolism Away From Oxidative Phosphorylation

Initially, metabolically quiescent naïve CD4 T cells rely on OxPhos to meet all their metabolic needs (18) (**Figure 1**). During activation, T cells undergo a shift in metabolism away from OxPhos by increasing glycolysis. T cell activation requires three main signals from antigen-presenting cells (APC); antigen presentation on major histocompatibility complex (MHC), co-stimulation, and cytokines/reactive oxygen species (ROS) (16, 19). CD4 T cell activation begins with an interaction between the T cell receptor (TCR) and peptide loaded on MHC class-II which initiates metabolic reprograming on naïve CD4 T cells (20). Co-stimulatory receptors present on both APCs and T cells will provide the second signal necessary for T cell activation. Finally, cytokines and ROS present in the local environment will provide a proinflammatory third signal to begin T cell differentiation toward specific effector responses (16, 21). Each signal lays the foundation for potential T cell fates and drives the shift in metabolism.

**Figure 2:**
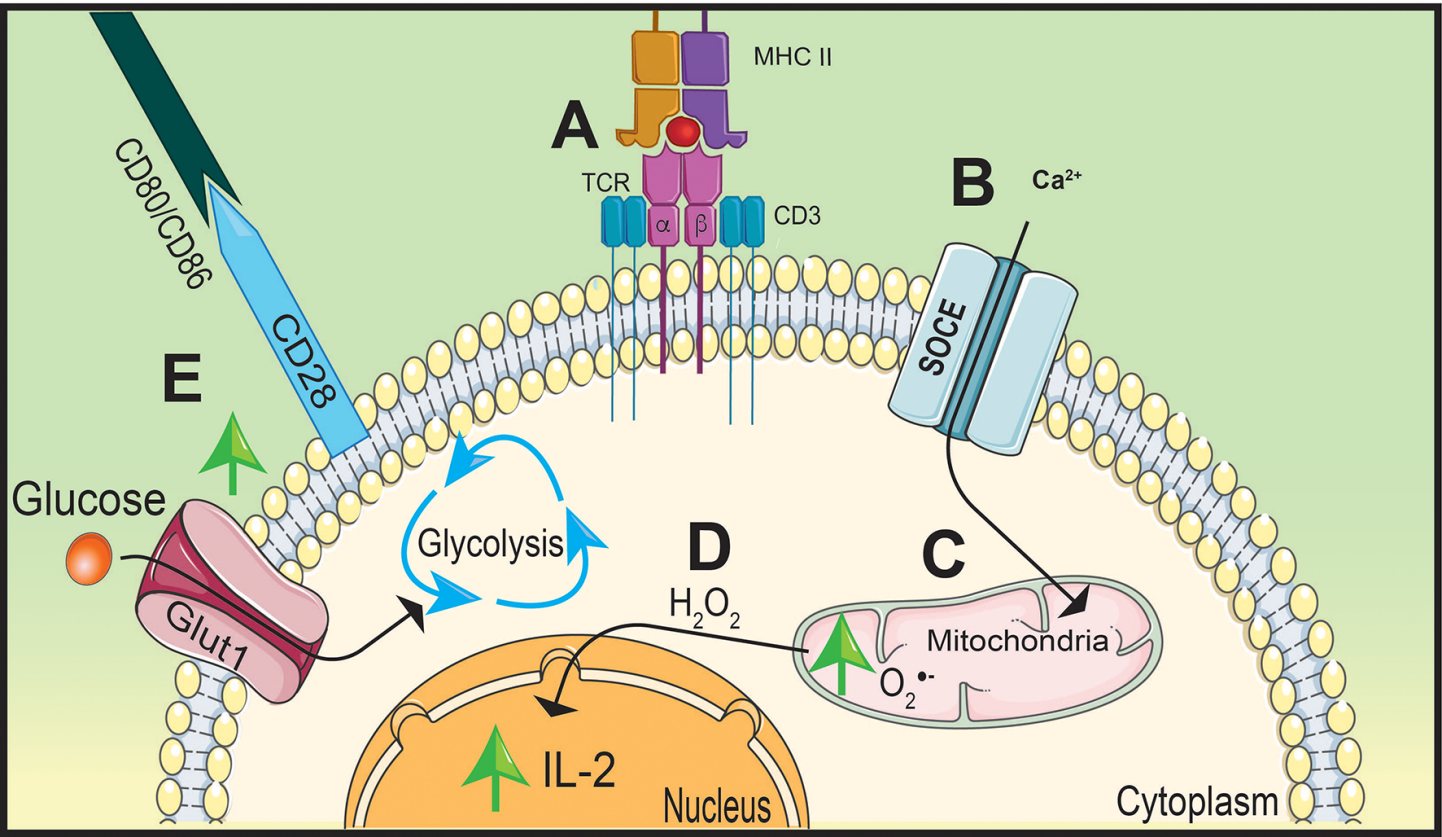
**Stimulation of TCR and CD28 causes a metabolic shift in naïve CD4 T cells.** T cell receptor (TCR) stimulation by peptide presented on major histocompatibility complex-II (MHC-II) **(A)** will increase calcium (Ca^2+^) entry into the cytoplasm through store operated calcium entry (SOCE) channels **(B)** increasing mitochondrial-derived superoxide $\left(O_{2}^{\cdot -}\right)$ generation **(C)**. Oxidative phosphorylation (OxPhos) will promote interleukin (IL)-2 expression by hydrogen peroxide (H_2_O_2_) signaling **(D)**. Simultaneously, CD28 will upregulate Glut1 expression on the cell membrane increasing glucose uptake shifting metabolism away from OxPhos to glycolysis to support rapid proliferation **(E)**.

TCR stimulation increases hydrogen peroxide signaling to support the upregulation of interleukin (IL)-2 (22, 23). The TCR is comprised of α and β chains and CD3 accessory chains that cumulatively bridge extracellular antigen presentation by APCs to facilitate into intracellular signaling (19, 24). After TCR stimulation, there is an influx of CD3-dependant store-operated Ca^2+^ entry (SOCE) that induces the accumulation of mitochondria near the TCR (23) (**Figures 2A, B**). Ca^2+^ influx into localized mitochondria influences IL-2 secretion by impacting the production of hydrogen peroxide (22) (**Figures 2C, D**). Due to its ability to stimulate differentiation and proliferation of T cells, production of IL-2 is a key cytokine for T cell activation (24). Deficiency in SOCE channels, such as Ca^2+^ release-activated Ca^2+^, results in a lack of IL-2 production that is potentially due to decreased hydrogen peroxide synthesis (25).

While Ca^2+^ influx is important to promote IL-2 production, the essential signaling molecule is hydrogen peroxide. This concept is supported by Ca^2+^-independent nuclear factor kappa-B (NF-κB) signaling, shown to be activated by hydrogen peroxide, significantly inhibiting IL-2 promoter regions as a result of blocking Ca^2+^ and/or ROS synthesis (26, 27). Similar to NF-κB signaling, hydrogen peroxide also mediates nuclear factor of activated T cells (NFAT) nuclear translocation and interaction with AP-1 to induce IL-2 production (28). These reports highlight the importance of Ca^2+^ influx and mtROS production to facilitate IL-2 production and T cell activation. However, optimal T cell activation also requires co-stimulatory molecule interactions in order to induce multiple signaling pathways and shift metabolism away from OxPhos to initiate T cell effector differentiation (29).

Co-stimulatory molecule, CD28, will increase glycolytic activity within the T cell to promote rapid proliferation (30, 31). Engagement of the TCR with the MHC-II/peptide complex forms an immunological synapse between the APC and the CD4 T cell by recruiting co-stimulatory molecules organized on lipid rafts on the cell membrane (32). This includes CD28 on the T cell which will interact with CD80 and CD86 on the APC (19). In order to maximize glycolytic potential, activated T cells will upregulate glucose transporter 1 (Glut1) to the cell membrane following co-stimulation and engage in aerobic glycolysis to ensure glucose will be utilized despite the presence or availability of oxygen (33, 34) (**Figure 2E**). CD28 will also upregulate signaling pathways that support T cell differentiation and glycolysis (31). One key pathway that is essential for T cell activation and proliferation is mitogen-activated protein kinase (MAPK) (30).

Within this pathway, extracellular signal-regulated kinase (ERK) signaling can regulate cell proliferation and differentiation by promoting glycolysis, facilitating the switch from OxPhos (35). While TCR stimulation will initiate ERK signaling in T cells, CD28 influences ERK activation by recruiting the appropriate kinases, such as lymphocyte-specific protein tyrosine kinase to enhance the signaling cascade (31). ERK phosphorylation in naïve T cells negatively regulates TCR-induced Ca^2+^ influx reducing OxPhos and promoting glycolysis (36). High glucose and glycolytic activity within the cell will support ERK/MAPK signaling, increase cell proliferation, differentiation, and prevent apoptosis (30, 37). The kinetics of T cell activation would be greatly diminished without CD28-dependent effects on ERK signaling and may result in weakened T cell responses (31).

In order to ensure a strong T cell response, CD28 stimulation also increases expression of pro-survival proteins including BCL-XL to prevent cell death in rapidly expanding T cells (38). Under non-apoptotic conditions, including T cell activation and expansion, overexpression of BCL-XL will result in mitochondrial depolarization and halting OxPhos (39). BCL-XL is able to influence OxPhos by reducing intracellular Ca^2+^ channel type 1 inositol 1,4,5-triphosphate receptor, which decreases the magnitude and duration of Ca^2+^ release following TCR signal transduction (39). BCL-XL will also redirect cytosolic Ca^2+^ away from the mitochondria through ER sequestration (39). Once co-stimulation has induced a metabolic state that is balanced between OxPhos and glycolysis; cytokines and ROS present in the immune environment will prime activated T cells for differentiation into specific effector subsets and responses.

The efficacy of both TCR and co-stimulatory signaling is greatly influenced by cytokines and ROS present during T cell activation (16, 21). Cytokine signaling can function in a paracrine and autocrine manner to influence APC and T cell activation (40). Downstream signaling pathways such as mTOR, NFAT, and NF-κB involved in T cell activation and differentiation are not only altered by the concentration of cytokines, but also by their kinetic expression as well (13, 21). Prior to activation, IL-7 is an essential cytokine for naïve T cell survival and homeostasis due to its inactivation of pro-apoptotic proteins (41, 42). Upon stimulation, T cells will secrete IL-2 to promote expansion and prime T cells for differentiation (41). An autoreactive Th1 effector response will result from a cytokine environment comprised of IFN-γ, IL-1β, TNF-α, IL-6, and IL-12 (43). IL-17, IL-23, and IL-21 cytokines result in an autoreactive Th17 effector response; while TGF-β and IL-10 will result in the promotion of self-tolerance (43).

Traditionally it was thought that cytokines were solely responsible for driving T cell differentiation toward specific effector responses. However, superoxide may also be contributing to differentiation by activating redox-dependent signaling pathways associated with certain T cell effector functions (44). Past research has demonstrated that even under conditions which utilized cytokines to skew toward a proinflammatory response, the reduction of ROS results in decreased NF-κB-regulated proinflammatory cytokine production (45). Additionally, the decrease in ROS, specifically superoxide, in both APC and T cells not only decreased proinflammatory IFN-γ synthesis, but also T-bet expression as well (16). These data accentuate the indispensable role of ROS generation in effector T cell responses.

## Mitochondrial Dysfunction Is Common Throughout T Cell Mediated Diseases

Due to the role of ROS in cellular homeostasis, irregular ROS generation contributes to disease pathology making it a prime target for metabolic reprograming therapies (46). ROS including superoxide are mainly generated by the mitochondria, but may also be produced by NADPH oxidase enzymes (47, 48). Antioxidants present within the cell and extracellular environment regulate ROS to prevent damage to the cells caused by oxidative stress (49). Oxidative stress and mitochondrial dysfunction are common pathologies normally found in T cell-mediated autoimmune diseases (49, 50), indicating that increasing our understanding of oxidative stress and ROS regulation may have potential for the development of novel therapies to mitigate autoimmune dysfunction.

**Figure 3:**
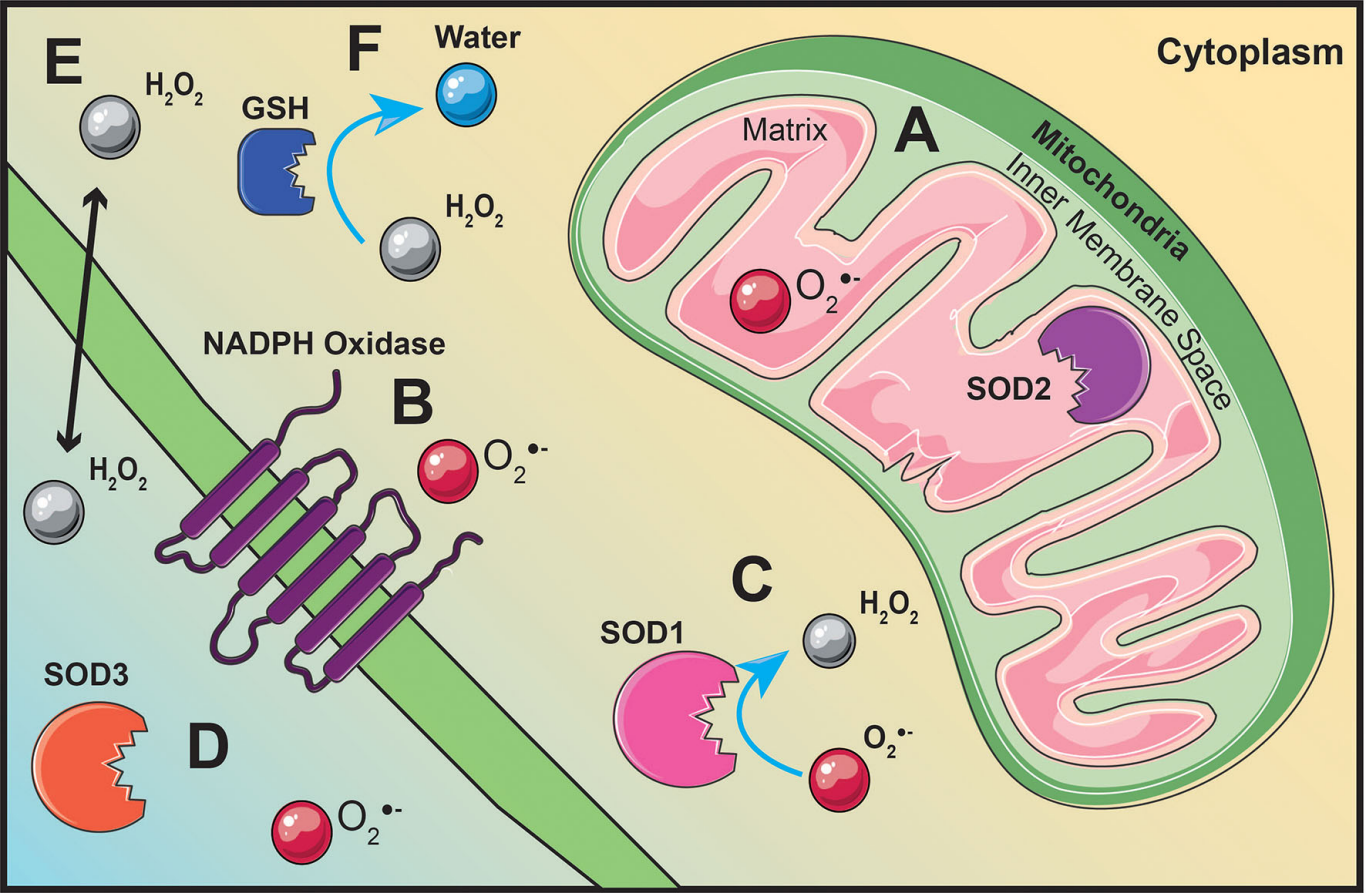
**ROS production originates from various locations throughout the cell.** Superoxide is generated within the mitochondrial matrix **(A)** or by membrane bound NADPH oxidases **(B)**. Superoxide dismutase (SOD) located within the mitochondrial matrix **(A)**, within the cytoplasm **(C)**, or extracellularly **(D)** will convert superoxide into hydrogen peroxide (H_2_O_2_) **(C)**. Hydrogen peroxide is able to act as signaling molecule within the cell or extracellular due to its ability to diffuse across the cell membrane **(E)**. Glutathione (GSH), located throughout the cytoplasm, will regulate hydrogen peroxide levels by converting hydrogen peroxide into water **(F)**.

ROS are generated throughout the cell as a result of various redox reactions (47, 48). The mitochondria are one of the main cellular sources of ROS synthesis due to electrons leaking from the election transport chain (ETC) and interacting with oxygen to generate superoxide (47) (**Figure 3A**). Pyruvate generated from glycolysis will migrate into the mitochondrial matrix, be converted to acetyl-CoA, and feed into the citric acid cycle (CAC) (51). Succinate dehydrogenase, also known as complex II, is the only membrane-bound component of the CAC and a complex within the ETC (52). Electron movement creates a proton gradient between the mitochondrial matrix and the inner membrane space driving the phosphorylation of adenosine diphosphate to adenosine triphosphate (ATP) by ATP synthase (53). Superoxide generation is not limited to the mitochondria, it can also be generated by NADPH oxidase enzymes on the plasma membrane (48) (**Figure 3B**).

Excess levels of superoxide can be toxic to the cell; therefore, superoxide dismutase (SOD) enzymes act to dismutate superoxide into hydrogen peroxide and molecular oxygen (46). There are three different isoforms of SOD located within and outside the cell. Copper- and zinc-containing SOD (Cu/Zn SOD, SOD1) is mainly localized in the cytoplasm (54) (**Figure 3C**). Manganese-containing SOD (MnSOD, SOD2) resides within the mitochondrial matrix (54) (**Figure 3A**). Similar to SOD2, extracellular SOD (SOD3) also contains manganese, except it is localized outside the cell (54) (**Figure 3D**). Regardless of its location, enzymatic regulation of superoxide by SOD will generate hydrogen peroxide as a result of its reaction (48) (**Figure 3C**). Unlike superoxide, hydrogen peroxide is stable, diffusible, and can regulate redox-dependent signaling pathways (55) (**Figure 3E**). Hydrogen peroxide can modulate pathway activation by inhibiting phosphatases, activating tyrosine kinases, and transcription factor activation (55). Catalase, glutathione peroxidase, and thioredoxin peroxidase are the main enzymes involved in maintaining hydrogen peroxide levels for optimal cell function and signaling (56).

A common factor in several mitochondrial-related diseases such as diabetes, obesity, and neurodegenerative diseases is oxidative stress (50). Oxidative stress is defined by an imbalance of ROS and antioxidant activity that could lead to cell and tissue damage (49). Excess levels of mtROS, specifically superoxide, can lead to cell damage and mitochondrial-dependent apoptosis (57). Under oxidative stress, a pore is created in the inner mitochondrial membrane, halting OxPhos, leaking cytochrome C into the cytoplasm, and inducing apoptosis as a result of DNA fragmentation and ultimately ending in cell death (57, 58). However, not all ROS production is detrimental to cells, as low levels of hydrogen peroxide can activate pro-survival proteins such as Bcl-2 (57).

Mitochondrial dysfunction and the generation of oxidative stress could contribute to disease pathology in SLE, T1D, and RA (4, 50, 59–61). Increased antioxidant activity in patients with SLE improved mitochondrial metabolism, decreased mtROS, and reduced inflammation (59). In T1D, ROS synthesis can influence autoreactive T cell activation and directly mediate β-cell damage partly due to weak antioxidant defenses in β-cells (60). An inadequate antioxidant defense may contribute to oxidative stress within the β-cell, influencing its dysfunction and immunogenicity in T1D (62). Oxidative stress due to mitochondrial dysfunction could also enhance inflammatory responses in RA (61). Analysis of genes and protein-protein interactions in human patients with RA revealed that numerous mitochondrial-related pathways influence RA pathogenesis (61).

Mitochondrial DNA (mtDNA) deletions may manifest in a variety of syndromes, diseases, or phenotypes varying in severity (63, 64). mtDNA encodes for 13 proteins that are essential for OxPhos (64). In humans, it was revealed that the single nucleotide polymorphism (SNP) nt13708A in mtDNA significantly increased susceptibility to MS and SLE (65, 66). This SNP is in the *MT-ND5* gene, which encodes a subunit of complex I in the ETC (65, 66). While both studies identified nt13708A, the functional consequences of this SNP remain unknown. However, not all mtDNA SNPs result in increased disease susceptibility. In T1D, the C5173A SNP in the *mt-ND2* gene provided protection against disease development (67). Further characterization of *mt-Nd2^a^*, an allelic variant of *mt-Nd2*, exhibited a reduction in ROS generated from complex I (67). These findings demonstrate that SNPs in mtDNA can modulate disease by altering OxPhos. Further studies on the effects of mtDNA deletions on OxPhos or superoxide generation could provide insight into the development of autoimmunity as a result of mitochondrial dysfunction.

Understanding the redox-dependent effects on immunometabolism and the generation of proinflammatory T cell responses needs to be further defined as a potential therapeutic strategy. The field of immunometabolism will define the molecular and cellular contributors that connect metabolism and immune cell function (14). Decades of research have shown nutrient availability and its effects on immune responses. Yet, only the last decade has provided insight on how metabolism coupled with ROS is a determinant of adaptive immune cell phenotype and effector responses.

## Differentiation of Th1/Th17 T Cells Is Promoted by Glycolysis and Stabilized by ROS

Inflammatory responses prime Th1/Th17 differentiation by promoting a metabolic environment toward glycolysis. An inflammatory response often creates an environment depleted of nutrients and oxygen (68). Coupled with proinflammatory cytokines that will induce the activation of TF necessary for Th1/Th17 differentiation, the lack of oxygen results in increased glycolysis and skewed T cell differentiation (69–71). In addition to an enhanced glycolytic environment, ROS synthesis will stabilize RORγt TF expression to promote Th17 differentiation (72, 73). Increasing our understanding of the effects of glycolysis and ROS synthesis in Th1 and Th17 CD4 T cell differentiation could give rise to novel targets for delaying T cell-mediated autoimmune diseases.

Th1 induction begins with APCs secreting IL-12, a key cytokine in Th1 differentiation, to upregulate proinflammatory cytokine IFN-γ secretion and TF T-bet expression (74). Deficiency in IL-12 will reduce autoreactive Th1 responses, including IFN-γ production, as a result of disrupting a feedback loop in activated T cells (75). The induction of this feedback loop is key to Th1 differentiation since IFN-γ is a proinflammatory cytokine that will upregulate other cytokines necessary for Th1 differentiation including IL-12 (70, 71).

Th1 differentiation is primarily driven by an upregulation of glycolysis to support expression of T-bet and IFN-γ (69–71). Differentiating T cells engaged in aerobic glycolysis preferentially translate *Ifng* mRNA *versus* those using OxPhos (76). Pyruvate, generated by glycolysis, is utilized by lactate dehydrogenase A, which also supports aerobic glycolysis in activated T cells in order to promote proinflammatory IFN-γ production (69). This suggests that glycolytic upregulation will promote proinflammatory cytokines that shift naïve CD4 T cell activation toward Th1 differentiation (69–71). Therefore, it is not surprising that cytokine-producing Th1 cells are diminished when the inability to transport glucose for glycolysis is facilitated by a Glut-1 deficiency (77). In T1D, there is an abundance of glucose, which could be contributing to an environment that preferentially drives Th1 differentiation.

IFN-γ production by autoreactive Th1 cells is involved in the destruction of insulin-secreting β-cells in T1D (78) (**Figure 1**). During insulitis, APCs infiltrate islets and migrate to peripheral lymphoid tissues where both naïve autoreactive CD8 and CD4 T cells are activated (9). Following activation, T cells migrate back to the islets located within the pancreas (79). Once in the pancreas, activated Th1 cells produce IL-2 and IFN-γ to mature cytotoxic CD8 T cell responses (80). The presence of other cytokines, such as IL-1β, TNF-α, and IFN-γ, promote Th1 differentiation in addition to inhibiting insulin secretion and inducing β-cell apoptosis (81, 82). Although both CD8 and CD4 T cells contribute to β-cell destruction, a deficiency of CD4 T cells protects against spontaneous T1D development in NOD mice (83). Therefore, reducing glycolysis and/or ROS production in autoreactive Th1 CD4 T cells represents a potential therapeutic strategy for delaying T1D.

Insulin is a key hormone that is secreted from β-cells in response to high glucose, impacting metabolism by signaling cells to uptake glucose and undergo glycolysis (84). Insulin signaling requires insulin-induced hydrogen peroxide generation to maintain autophosphorylation of insulin-stimulated insulin receptor (73). Inhibition of superoxide generation by complex II of the ETC prevents hydrogen peroxide generation after insulin stimulation, disrupting insulin signaling and glucose uptake (85). Decreasing glycolytic signaling in T cells by regulating hydrogen peroxide to decrease glucose uptake may be another immunotherapy used to orchestrate a metabolic blockage. The use of the glycolytic inhibitor 2-deoxy glucose (2-DG), has shown promise in diminishing autoreactive CD4 T cell responses without harming other aspects of the immune system (34). This would be beneficial compared to other potential treatment options, such as rapamycin, which acts as a universal immunosuppressant targeting APCs and T cells (86). Rapamycin is a mTOR inhibitor that would prevent all cells from being able to monitor for DNA damage, synthesize proteins, and coordinate an appropriate response to intracellular stress if administered systemically (87). This could alter immune function, lipid homeostasis, and muscle mass homeostasis resulting in increased susceptibility to other diseases (87). Therefore, there is a need for the development of targeted metabolic therapies specific for autoreactive T cells without impairing the ability of the immune system to mount defenses against microbial pathogens.

Th17 cells, like Th1 cells, also rely on glycolysis for their differentiation. Currently, there are studies showing the promise of this type of metabolic targeting therapy in Th17-mediated diseases including MS (88). Suppression of glycolysis *via* deletion of Glut1 has been shown to protect mice from Th1 and Th17 cell-driven diseases such as colitis and inflammatory bowel disease by decreasing the expansion and survival of T cells (77). A possible target for diminishing glycolysis in Th17 cytokine responses would be decreasing hypoxia-inducible factor (HIF)-1α expression. HIF-1α is a TF that will contribute to metabolic programming of activated T cells by mediating a switch from OxPhos to aerobic glycolysis (89, 90). HIF-1α will specifically stimulate Th17 differentiation by binding to the RORγt promoter to increase IL-17A production (89). Prostaglandin E2 (PGE_2_) will boost glycolytic activity by stabilizing HIF-1α (91), while simultaneously skewing T cells away from a Th1 phenotype by inhibiting IFN-γ production (92). Prevention of hypoxic environments that will foster glycolytic activity *via* PGE_2_ inhibition is a current treatment option for Th17-mediated autoimmune diseases including RA (92).

In MS, pathogenesis is driven by pathogenic Th17 cells interacting with the central nervous system (CNS) (93) (**Figure 1**). Naïve T cells are primed outside the CNS by APCs, cross the blood-brain barrier, and produce proinflammatory responses that result in the destruction of myelin and axons (93). Experimental autoimmune encephalomyelitis (EAE) is a mouse model used to study MS and studies have shown that increased ROS during pathogenic Th17 differentiation is another possible metabolic target for MS (94). Inflammatory Th17 responses are promoted by hydrogen peroxide stabilizing insulin receptor signaling to activate HIF-1α (72, 73). T cells deficient in insulin receptor signaling will have a diminished inflammatory response when activated, demonstrating the importance of insulin receptor stabilization by hydrogen peroxide for pathogenic Th17 differentiation (72, 95). Additionally, hydrogen peroxide contributes to Th17 differentiation by activating HIF target genes and stabilizing HIF-1α proteins (96). By inhibiting mitochondrial OxPhos during Th17 activation, mTOR signaling and the expression of basic leucin zipper transcriptional factor ATF-like (BATF) will be decreased (97). Without BATF to increase chromatin accessibility of Th17 transcription factors, mice displayed a resistance to EAE without affecting Th1/Th2 effector responses (98). This observation highlights the promise of metabolic therapies targeting ROS production in autoreactive Th17 cells, but immunometabolic reprogramming can also affect Treg differentiation as another potential avenue to delay EAE disease onset (97).

## OxPhos Is Required for Treg/Th2 Differentiation and Treg Suppressive Function

It has been debated whether the loss of Treg function and/or the number of Treg cells is a major contributor in T cell-mediated autoimmune diseases such as T1D, MS, and RA (99). Tregs have a broad set of functions including ensuring tolerance to autoantigens, limiting excess immune responses, and providing homeostasis of various tissues (100). Their development is similar to conventional T cells, but they are unique in their underlying self-reactivity during thymic selection, allowing them to suppress other CD4 T cell effector populations (2, 101). Strategies to increase the number of Tregs and enhance immunosuppressive Treg function are ideal approaches to restore peripheral tolerance in autoimmune diseases.

Therefore, studies to determine how immunometabolism contributes to Treg homeostasis, differentiation, and function are worthy research endeavors. Loss of the anti-apoptotic factor, Bcl-2, did not alter the quality or quantity of Tregs isolated from mice (102). However, increased expression of Bcl-2 by IL-7 enables natural Tregs to survive and proliferate properly while they circulate between secondary lymphoid organs (100). Bcl-2 is known to increase mtROS superoxide production in complexes I and III within the ETC (103). Tregs deficient in complex III-derived superoxide are able to maintain stable Foxp3 expression, cell proliferation, and survival, yet their suppressive capacity is lost (104, 105). This paralleled loss of function emphasizes the importance of proper mitochondrial metabolism throughout T cell activation and homeostasis.

There is a close developmental relationship between Treg and Th17 cells. TGF-β alone will induce Treg differentiation, however if IL-6 is present, Th17 cells will be preferentially induced (5). IL-6 will promote glucose uptake and metabolism (106, 107). This is due to IL-6 terminating Foxp3-mediated inhibition of Th17 TF RORγt (107). Foxp3 is a lineage-specific TF for Treg cells (3) (**Figure 1**). In the absence of other proinflammatory cytokines, Foxp3 inhibits Th17 differentiation by blocking RORγt activity in activated T cells (108). IL-2 negatively regulates IL-17 production while driving Treg proliferation, differentiation, and function (109, 110). In SLE, due to the reduction of IL-2, there is a decrease in the Treg population (111). In the absence of HIF-1α, a Th17 promoting factor, Th17 cytokine responses will be dampened and Foxp3 Treg differentiation will be elicited instead (97). This could be due to the absence of the Foxp3 ubiquitin-mediated degradation pathway that is regulated by HIF-1α (112). HIF-1α increases RORγt expression while simultaneously tagging Foxp3 protein for ubiquitination (89).

Myelocytomatosis oncogene (Myc) is a TF that drives metabolic reprogramming toward glycolysis (34). Expression of Foxp3 suppresses glycolysis by binding to the Myc promotor and suppressing Myc gene expression (34). Diminishing glycolysis *via* Glut1deficiency does not affect Treg differentiation or cell numbers (77) since Treg cells utilize fatty acid oxidation (FAO) for Treg differentiation (113). By suppressing glycolysis, Foxp3 reactivates OxPhos, which is essential for Treg metabolism, stability, and function (**Figure 1**).

Similar to Tregs, Th2 cells upregulate fatty acid lipid metabolism and inhibition of these metabolic pathways will reduce a Th2-mediated response (114). The key role of OxPhos in Th2 differentiation is highlighted by a study that showed that reduced mtROS produced by complex-I inhibited anti-CD3-induced IL-2 and IL-4 expression and stunted Th2 differentiation (27). The production of IL-4 aids in the differentiation of naïve T cells toward the Th2 phenotype by inducing expression of the key TF Gata3 (8, 115). Expression of Gata3 is sufficient to induce the Th2 phenotype because it creates a positive feedback loop by inducing IL-4, which maintains Th2 cell identity (114). Increased activity of the kinase mTOR is present in all effector Th lineages in order to coordinate cell proliferation and metabolism (87). Unique to Th2 cells is the increase in fatty acid uptake by peroxisome proliferator activated receptor gamma (PPAR-γ) that also regulates fatty acid metabolism (114). Both IL-4 and mTOR promote PPAR-γ in Th2 cells and its absence will not only affect OxPhos, but Th2 function as well (114). Unfortunately, Th2 differentiation is diminished in individuals taking rapamycin due to its inhibitory effects on mTOR activity (87). Loss of Th2 function *via* decreased mTOR could be associated with decreased PPAR-γ binding to key genes such as *Gata3, Il5*, and *Stat5* (116).

Th2 cells protect from parasitic helminths infections and stimulate repair of damaged tissue repair (117). While there are no Th2-mediated autoimmune diseases, modulating the balance between Th1 and Th2 cells can affect autoimmune responses in animal models of RA (118). Treatment of arthritic mice with IL-4 suppressed disease, but did not reverse disease progression (118). Similar success was also observed in mouse models of T1D that skewed T cell responses toward a Th2 phenotype in an effort to prevent the activation of autoreactive Th1 cytokine responses (119). The efficacy of Th2 skewing has not been investigated in mouse models of MS or SLE. However, the loss of the nuclear factor erythroid 2-related factor 2 (NRF2) TF involved in inducing an antioxidant response, diminished Th2 cytokine responses and concomitantly, increased Th1 differentiation and SLE (120). While Th2 skewing may delay autoimmunity, Th2 cells also contribute to asthma and allergy. Allergen immunotherapy involves continuous exposure to allergens at increasing concentrations and is the only well-established treatment for asthma that targets Th2 cells (121). While the mechanism is still undefined, allergen immunotherapy has been shown to skew T cell differentiation away from a Th2 phenotype toward a Treg or Th1 phenotype (122).

## Decreasing Pathogenic CD8 T Cells in Autoimmunity by Promoting OxPhos

Similar to CD4 T cells, CD8 T cell activation requires antigen presentation, co-stimulatory molecule interactions, and the synthesis of cytokines and ROS (123, 124). However, unlike CD4 T cells, MHC-I is required for CD8 TCR stimulation, and deficiency in co-stimulatory molecules such as CD40L and CD28 does not result in anergy or decreased proliferation (125). Additionally, despite an increase in glycolysis to support clonal expansion, a deficiency in Glut1 expression does not affect CD8 T cell proliferation after activation (77, 126). Rather, their role in viral clearance is maximized by proinflammatory cytokines such as IFN-γ and IL-12, because it shifts activation toward short-lived effector cell (SLEC) populations (123).

The two main subsets of CD8 T cells are SLEC and memory precursor effector cells (MPEC) (123). Following TCR stimulation, mitochondrial fusion occurs in CD8 T cells to support increased OxPhos that promote cell proliferation (127, 128). Halting OxPhos *via* the deletion of AMP-activated protein kinase (AMPK) promotes the activation of the mTOR pathway and subsequently, defective CD8 T cell memory differentiation (129, 130). Inhibition of mTOR through AMPK activity improves the quality of CD8 memory T cells by accelerating effector to memory transition without affecting CD8 T cell effector function (129, 131). The effect of AMPK on CD8 T cell differentiation resulted in an interest in AMPK as a possible target for CD8 T cell memory generation. AMPK, in addition to inhibition of growth regulator mTOR, also regulates mitochondrial fission/fusion and promotes mitochondrial biogenesis (132). Due to memory T cells having an increased mitochondrial mass, AMPK regulation of mitochondrial biogenesis could contribute to increasing mitochondrial mass promoting memory T cells rather than cytotoxic CD8 T cells (133).

In T1D, APCs such as macrophages and dendritic cells are the first immune cells that infiltrate islets and contribute to pancreatic β-cell destruction (134). Macrophages activate Th1 CD4 T cells to produce IL-2 and IFN-γ to facilitate the differentiation of CD8 T cells to become cytotoxic (80). IL-1β secretion by APCs can function as a proinflammatory third signal for T cell maturation and enhance antigen-specific CD8 T cell effector function by increasing cytotoxic activity and IFN-γ production (135). If MHC-I is not expressed on β-cells, mice were protected from diabetes development (134). Therefore, strategies that can inhibit CD8 T cell effector responses would be beneficial as a T1D therapeutic. Recent investigations have tested the efficacy of 2-DG to inhibit glycolysis and autoreactive CD8 T cell cytolytic responses in T1D (136). Inhibition of glycolysis decreased islet infiltration of pathogenic CD8 T cells (136), but the effects of 2-DG on cytotoxic CD8 T cell effector responses were not determined.

In MS, CD8 T cells are a viable target for therapy (137). Pathogenic CD8 T cells have been located in MS plaques, cerebrospinal fluid, and demyelinated axons (137). Mutations in MHC-I, along with gradual upregulation of MHC-I on various cell types throughout disease progression are associated with increased risk for MS and may also contribute to the increased ratio of CD8 to CD4 T cells in MS brain lesions (138). The accumulation of the glycolytic byproduct, lactate, in the cerebrospinal fluid of MS patients compared to controls has recently been considered an indicator of disease progression (139). Promoting a metabolic shift to decrease glycolytic activity may provide protection from disease progression. Lactic acid is able to promote OxPhos by inducing glycolysis/OxPhos interconversion to suppress cytotoxic CD8 T cell proliferation and cytokine production (140, 141). These biomarkers and different metabolic indicators for disease may all be therapeutic options worth experimentally perusing in order to decrease pathogenic CD8 T cell activity.

## Decreased Antioxidant Activity in T Cell-Mediated Diseases Can Be Utilized as a Biomarker or Therapy for Autoimmunity

Antioxidants are effective in decreasing oxidative stress and loss of antioxidant activity may also be involved in autoimmune diseases. Glutathione (GSH) is a tripeptide antioxidant consisting of glutamate, cysteine, and glycine, located within the cytoplasm and utilized by the cell to detoxify ROS such as hydrogen peroxide (142, 143) (**Figure 3F**). The reductive capability of the sulfhydryl group contained within the cysteine makes GSH a pivotal component of protecting the cell from oxidative damage and stress resulting from excess ROS (143). Decreased activation of the NRF2 pathway, which regulates antioxidant genes including GSH, is observed across many T cell-mediated diseases (144). This is most likely due to GSH supporting activation-induced metabolic reprograming in T cells. By decreasing ROS, GSH reinforces glycolytic metabolic reprogramming that is required by inflammatory Th1 and Th17 cytokine responses (145). Studies have also shown that a deficiency in GSH results in impaired IFN-γ and IL-17 cytokine production (146).

Hyperglycemia is associated with increased oxidative stress, free radical production, suppression of antioxidant function, and is also a common complication in T1D and type 2 diabetes (T2D) (49, 147, 148). Furthermore, increased cytosolic hydrogen peroxide generation in pancreatic islets as a result of decreased GSH and SOD in individuals with T2D leads to impaired insulin secretion (149, 150). Children diagnosed with T1D show significantly decreased GSH antioxidant capacity compared to age-matched healthy children (147, 151). Therefore, it is encouraging that NRF2 activators have shown a great deal of promise as a therapeutic in pre-clinical trials for diabetic complications associated with increased oxidative stress (152).

GSH deficiency is also prevalent in MS (153). Compared to adjacent white matter, there is a significant reduction in GSH in MS plagues (153). GSH delivery to the CNS is difficult due to the inability to cross the blood-brain barrier, short half-life, and the requirement of high doses to achieve a therapeutic effect (154). While oral GSH did not impact disease progression, there are clinical trials to investigate NRF2 activation as a potential therapeutic in MS to increase downstream expression of antioxidants such as GSH (154). A phase 3 clinical trial for MS demonstrated that oral treatment with delayed-release dimethyl fumarate (DMF) was effective in activating NRF2, but further studies are needed to determine if GSH levels were increased and if MS could be delayed following DMF treatment (155).

In mouse models of RA, overexpression of SOD3 suppressed proinflammatory cytokine production and disease development (156). Unfortunately, human translation is limited due to a short half-life and instability of SOD (157). Interestingly, saliva taken from individuals with SLE displayed decreased presence of SOD compared to healthy controls (158). Therefore, it has been proposed that decreased SOD activity could alternatively be utilized as a biomarker for SLE (158). Other investigators have employed SOD mimetics in mouse models of T1D to increase stability and longevity of antioxidant activity in an effort to delay pancreatic β-cell destruction (159). The use of SOD mimetics can delay the adoptive transfer of T1D with a diabetogenic CD4 T cell clone that was partly due to a decrease in T cell proliferation and IFN-γ production (160). Similar results were also observed when CD8 T cells were treated with a SOD mimetic resulting in decreased proliferation, cytokine production and cytolytic effector responses (124). The ability of SOD mimetics to delay T1D progression and inhibit autoreactive T cell responses suggests these novel antioxidants may also be applicable for the treatment of SLE and RA in future studies.

## Conclusion

Increasing our understanding of the role of immunometabolism in T cell-mediated autoimmune diseases may provide novel insights into the redox-dependent mechanisms involved in immune dysregulation. Specifically, determining the homeostatic role of glycolysis and OxPhos in naïve and effector T cells may bridge the knowledge gap of metabolic reprogramming in T cell activation and differentiation. T1D, MS, and RA are autoimmune diseases that would benefit from metabolic reprogramming of autoreactive T cells to alleviate disease pathogenesis. Metabolically targeting glycolysis to decrease Th1 cytokine responses in T1D may prevent T cell-mediated β-cell destruction. Similarly, inhibiting glycolysis by targeting HIF-1α stabilizers such as hydrogen peroxide and PGE_2_ can also dampen Th17 cytokine responses in MS to delay neuronal damage. Decreasing glycolysis is not only effective in limiting autoreactive CD4 T cell responses, but can also diminish cytotoxic CD8 T cell effector responses in T1D and MS. Increasing our knowledge of the contribution of mtROS in T1D, MS, RA, and SLE and the effect of antioxidants to diminish mtROS may provide a novel immunotherapeutic approach to limit T cell-mediated autoimmunity in the near future.

## Author Contributions

MC and HT conceived, wrote, and edited the manuscript. All authors contributed to the article and approved the submitted version.

## Funding

This work was supported by an NIH/NIDDK R01 DK099550, JDRF award SRA-2016-270-S-B, JDRF award 2-SRA-2019-692-S-B, NIH/NIDDK R56 DK126456, and NIH/NIDDK R01 DK127497.

## Conflict of Interest

The authors declare that the research was conducted in the absence of any commercial or financial relationships that could be construed as a potential conflict of interest.
